# Survival in sporadic ALS is associated with lower p62 burden in the spinal cord

**DOI:** 10.1093/jnen/nlad051

**Published:** 2023-07-06

**Authors:** Monica Pinkerton, Guinevere Lourenco, Maria Torres Pacheco, Glenda M Halliday, Matthew C Kiernan, Rachel H Tan

**Affiliations:** Brain and Mind Centre, University of Sydney, Sydney, New South Wales, Australia; Faculty of Medicine and Health, School of Medical Sciences, University of Sydney, Camperdown, New South Wales, Australia; Brain and Mind Centre, University of Sydney, Sydney, New South Wales, Australia; Faculty of Medicine and Health, School of Medical Sciences, University of Sydney, Camperdown, New South Wales, Australia; Brain and Mind Centre, University of Sydney, Sydney, New South Wales, Australia; Brain and Mind Centre, University of Sydney, Sydney, New South Wales, Australia; Faculty of Medicine and Health, School of Medical Sciences, University of Sydney, Camperdown, New South Wales, Australia; Brain and Mind Centre, University of Sydney, Sydney, New South Wales, Australia; Institute of Clinical Neurosciences, Royal Prince Alfred Hospital, Sydney, New South Wales, Australia; Brain and Mind Centre, University of Sydney, Sydney, New South Wales, Australia; Faculty of Medicine and Health, School of Medical Sciences, University of Sydney, Camperdown, New South Wales, Australia

**Keywords:** Amyotrophic lateral sclerosis, Motor neurons, p62 pathology, pTDP-43

## Abstract

The autophagy marker p62 appears as a consistent component of pathological aggregates in amyotrophic lateral sclerosis (ALS) and its modulation to facilitate protein degradation has been proposed as a potential therapeutic target. Importantly, recent studies have implicated diffuse phosphorylated TDP-43 inclusions that are immuno-negative for p62 in more rapid disease, highlighting the need for better understanding of p62 involvement in ALS pathogenesis. The present study set out to assess p62 pathology in the motor neurons of 31 patients with sporadic ALS that had either a short (<2 years) or longer (4–7 years) disease duration to determine its association with pTDP-43 pathology, motor neuron loss, and survival in sporadic disease. Our results identified significantly more cytoplasmic p62 aggregates in the spinal cord of patients with a shorter survival. Disease duration demonstrated a negative association with p62 burden and density of remaining motor neurons in the spinal cord, suggesting that survival in sporadic ALS is associated with the successful clearance of lower motor neurons with p62 aggregates. These findings implicate the autophagy pathway in ALS survival and provide support for further study of p62 as a potential prognostic biomarker in ALS.

## INTRODUCTION

Amyotrophic lateral sclerosis (ALS) is characterized by the loss of upper and lower motor neurons (1). p62 is a scaffold protein that facilitates the degradation of abnormal proteins through autophagy and the ubiquitin-proteasome system (2). It is a consistent component of the pathological phospho-TDP-43 (pTDP-43) inclusions in the motor neurons in ALS but whether its accumulation is neuroprotective, or a reflection of aberrant underlying mechanisms remains unclear (3–5). Interestingly, recent studies have identified a higher burden of pathological pTDP-43 in the lower motor neurons of patients with more rapid ALS (6), with this accumulation suggested to be driven by diffuse pTDP-43 inclusions that are negative for p62 (7). Given that the role of autophagy in ALS pathogenesis remains poorly understood, the present study set out to assess p62 aggregates in the motor neurons of patients with short compared to longer-duration ALS to determine its association with pTDP-43 pathology, motor neuron loss, and survival in sporadic disease.

## MATERIALS AND METHODS

### Ethics approval

This research project was approved by the Human Research Ethics Committee of the University of Sydney and complies with the statement on human experimentation issued by the National Health and Medical Research Council of Australia. Tissues were selected from a neuropathological series collected by the Sydney Brain Bank through regional brain donor programs in Sydney, Australia. The brain donor programs hold approval from the Human Research Ethics Committees of the South Eastern Sydney Area Health Services and comply with the statement on human experimentation issued by the National Health and Medical Research Council of Australia.

### ALS patients

All cases with a pathological diagnosis of ALS-TDP with a disease duration of either <2 years (referred to in this study as “short survival”) or 4–7 years (referred to in this study as “longer survival”) were selected from a neuropathological series collected by the Sydney Brain Bank through regional brain donor programs (8, 9). These donor programs hold approval from the Human Research Ethics Committees of the University of New South Wales. All ALS cases demonstrated upper and lower motor neuron degeneration accompanied by cytoplasmic TDP-43 inclusions (1). All cases had previously been staged for topographical progression of TDP-43 (10) and assessed for genetic mutations in the *C9ORF72*, *TARDBP*, and *SOD1* genes. Given the focus on pathology in disease progression of sporadic ALS, cases that had frontotemporal lobar degeneration, a family history of disease, or genetic mutation were not included. A total of 31 cases met these inclusion criteria and comprised 15 cases with a short disease duration (<2 years) and 16 with a longer disease duration (4–7 years). This research project was approved by the Human Research Ethics Committees of the University of Sydney and complies with the statement on human experimentation issued by the National Health and Medical Research Council of Australia.

### Quantitation of p62 and pTDP-43 pathologies in motor neurons

The upper motor neurons in the motor cortex and lower motor neurons in the hypoglossal nucleus and lumbar spinal cord are the predilection sites of ALS pathology (9, 10) and were assessed in the present study. Formalin-fixed, paraffin-embedded tissue blocks were available for a single hemisphere of the motor cortex and hypoglossal nucleus, and bilaterally for the anterior horns of the lumbar spinal cord. Two 10-μm sections were requested from these blocks on separate occasions for immunostaining with antibodies against phospho-TDP-43 (S409/410) (Cosmo Bio Co., TIP-PTD-M01, 1:80,000, [Tokyo, Japan]) and p62 (BD Biosciences, 610833, mouse, 1:250, [Franklin Lakes, NJ]). All slides were counterstained with hematoxylin for quantitation of neuronal populations. Slides were digitally scanned using the Olympus VS-120 slide scanner and each region-of-interest (ROI) was overlaid with a grid (comprised individual frames that each measured 315 μm×236 μm) for quantification at 40× magnification. The number of frames that fell within each ROI were counted and multiplied by the size of each frame to derive the area assessed. The total number of motor neurons as well as the number of motor neurons with p62 or pTDP-43 aggregates were counted and divided by the area in each ROI to derive the density of motor neurons and density of motor neurons with pathological inclusions. Quantitation was performed using the same protocol by 2 independent raters blind to case details and treatment group. There was an inter-rater variance of <5%.

### Statistics

Statistical analysis was performed using SPSS (Version 25) with a p value <0.05 taken as significant. Group differences were determined using one-way ANOVA for age, postmortem delay and ALS-TDP stage, and with chi-square test for gender and site of disease onset. Group differences were assessed using multivariate analysis. Correlation analyses were performed with Spearman rank correlation analyses.

## RESULTS

### Demographic data

As expected, disease duration was significantly lower in the short compared to longer disease duration group (p < 0.001; Table). However, the short- and longer-duration groups demonstrated no significant difference in age at death, postmortem delay, site of onset, sex, or ALS-TDP stage (p > 0.1 for all; Table).

**Table.** Demographic and clinicopathological data of the ALS cases (n = 31) with a disease duration of <2 years (short survival) or 4–7 years (longer survival) included in this study

	Short survival	Longer survival	p value
N (% male)	15 (67%)	16 (63%)	0.8
Age at death (years)	66 ± 2	70 ± 2	0.3
Disease duration (years)	1 ± 0.2	5 ± 0.2	<0.001
Postmortem delay (hours)	27 ± 5	26 ± 3	0.9
Prevalence UL/LL/Bulbar (%)	42/25/33	36/50/13	0.3
ALS-TDP stage (10)	2.8 ± 0.4	2.3 ± 0.3	0.3

Data for mean age, disease duration, postmortem delay, and ALS-TDP stage are presented as mean ± SE. All other data are presented as frequency of occurrence within the survival group.

UL, upper limb; LL, lower limb.

### Neuron density

**Figure 1.**
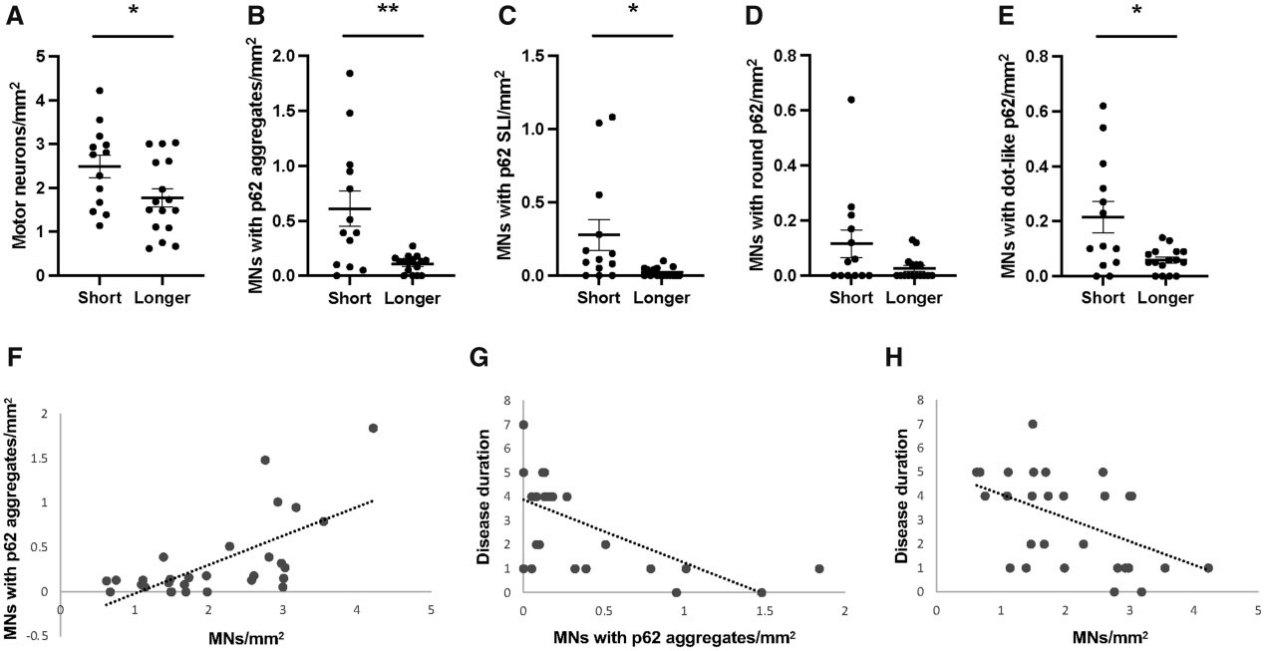
Density (mean ± SE) of motor neurons in the spinal cord **(A)** and density of motor neurons with cytoplasmic p62-immunoreactive aggregates in the spinal cord **(B–E)** of ALS cases with a short (<2 years) or longer (4–7 years) disease duration. A significant positive association was identified between the density of p62 aggregates and surviving motor neurons (MNs) in the spinal cord **(F)**. Increasing disease duration was associated with decreasing density of p62 aggregates **(G)** and surviving motor neurons in the spinal cord **(H)**. SLI, skein-like inclusions. *p < 0.05; **p < 0.005.

The density of remaining motor neurons in the spinal cord was significantly lower in the long- compared to the short-disease duration group (p < 0.05; Fig. 1). No significant differences in motor neuron density were identified in the motor cortex or hypoglossal nucleus (Supplementary Data  Table S1 and Fig. S1).

### p62 immunohistochemistry

**Figure 2.**
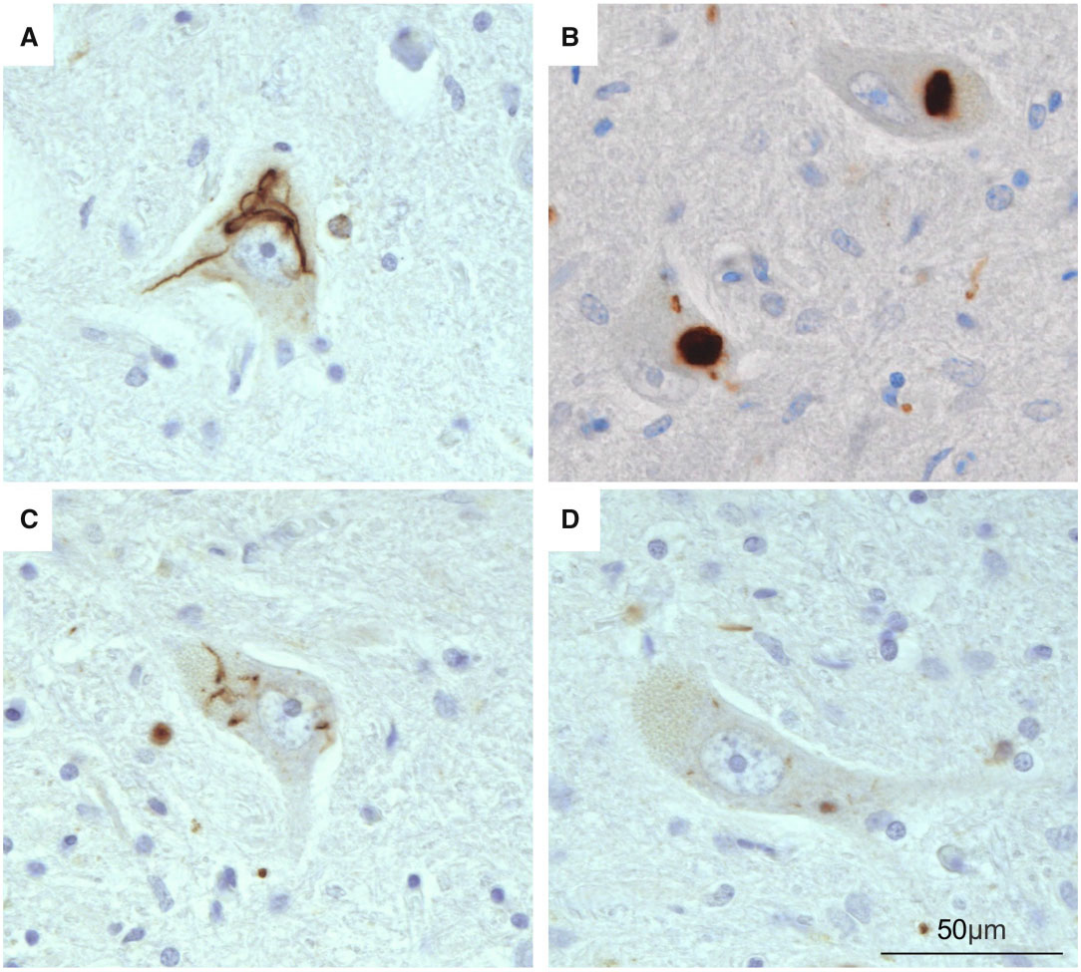
Morphology of cytoplasmic p62-immunoreactive inclusions in the lower motor neurons. **(A, C)** Skein-like inclusions **(B)**, dense round inclusion, and **(D)** dot-like inclusions.

Cytoplasmic p62 aggregates were identified in the lower motor neurons and showed skein-like, dense-round, or dot-like morphology (Fig. 2). Consistent with how upper motor neurons have been shown to demonstrate scarce diffuse cytoplasmic pTDP-43 (11), p62 aggregates were not identified in the upper motor neurons. A significantly greater burden of cytoplasmic p62 aggregates was identified in the spinal cord of the short- compared to longer-disease duration cases (Fig. 1). Comparison of p62 aggregate morphologies demonstrated that this difference was driven by skein-like aggregates in the spinal cord (p < 0.01; Fig. 1). No significant difference in the density of motor neurons or density of motor neurons with p62 inclusions were identified in the hypoglossal nucleus (Supplementary Data  Fig. S1).

### pTDP-43 immunohistochemistry

A significantly greater density of motor neurons with cytoplasmic pTDP-43 aggregates was identified in the spinal cord and hypoglossal nucleus of the short compared to the long survival group (spinal cord [mean±SEM] of 1.6 ± 0.3 in the short and 0.5 ± 0.1 in the longer group, p < 0.001; hypoglossal nucleus [mean±SEM] of 2.5 ± 0.6 in the short and 1.0 ± 0.3 in the longer groups, p < 0.02). Comparison of inclusion morphologies identified more skein-like pTDP-43 inclusions in the spinal cord of the short survival group (mean±SEM: 2.5 ± 0.6 in the short and 1.0 ± 0.3 in the longer group). No significant difference was identified in the upper motor neurons (Supplementary Data  Table S1).

### Correlation analysis

As expected, a significant positive correlation was found between cytoplasmic p62 and pTDP-43 aggregates (rho = 0.5, p < 0.001). Increasing p62 and pTDP-43 aggregates were associated with increasing density of motor neurons in the spinal cord (rho>0.7, p < 0.001 for both). Increasing disease duration was associated with decreasing cytoplasmic p62, pTDP-43, and motor neuron density in the spinal cord (rho>−0.6, p < 0.001 for all) (Fig. 1). No relationships between age at death and neuronal density or pathological burden were found (p > 0.1 for all).

## DISCUSSION

The present study assessed the autophagy marker p62 in the motor neurons of patients with sporadic ALS and identified significantly more cytoplasmic p62 aggregates in the spinal cord of patients with a short (<2 years) compared to longer (4–7 years) disease duration. As expected, a positive association was identified between p62 and pTDP-43. Both p62 and pTDP-43 demonstrated a negative association with motor neuron loss in the spinal cord as well as with disease duration, suggesting that survival in sporadic ALS is associated with the successful clearance of spinal motor neurons with pathological aggregates.

Although diffuse pTDP-43 inclusions that are immunonegative for p62 have been implicated in more rapid disease progression (7, 12), a greater proportion of pTDP-43 compared to p62 is well-recognized at autopsy (13, 14). This suggests that pathological pTDP-43 precedes ubiquitination and the recruitment of p62-mediated degradation of abnormal aggregates (2). Because the accumulation of p62 is considered an indicator of autophagy inhibition, the present findings of significantly more p62 aggregates in the spinal cord of patients with a shorter disease duration implicates deficits in downstream autophagosomal pathways in ALS survival (15). Our results demonstrate a significant negative correlation between disease duration and p62 accumulation, suggesting that a functioning autophagy pathway enables the successful clearance of motor neurons bearing pathological aggregates and longer survival. This is supported by studies in experimental models that have shown that p62 accumulates in response to molecular aggregates and disease progression is driven by a deficit in the clearance of pathological accumulation (16).

Modulation of p62 to promote clearance of pathology has been proposed as a potential therapeutic target for ALS (17, 18). However, p62 has also been implicated in aggregate formation (18, 19) with experimental models demonstrating counteracting roles in early and late disease (20). This highlights the complexity of the involvement of p62 in ALS pathogenesis and the need for further research. Future studies assessing other autophagy biomarkers such as LC3B, LC3A, beclin-1, ULK1, and VPS34 will enable further understanding as to whether survival and quality of life in ALS is associated with changes in autophagosomal formation and/or autophagy flux (17). This may elucidate potential therapeutic targets that improve survival in ALS. Importantly however, the prognostic value of p62 is well-recognized in cancer, where increased cytoplasmic p62 expression is associated with more aggressive tumor behavior and poorer prognosis (15,21–25). Elevated p62 levels have also recently been identified in the cerebrospinal fluid of patients with dementia (26), providing further support for the study of p62 as a potential prognostic biomarker in ALS.

In summary, the present study demonstrates significantly more p62 aggregates in the spinal cord of patients with shorter ALS survival. It demonstrates a significant negative correlation between p62 accumulation with neuron loss and disease duration thereby underscoring the importance of the autophagy pathway to survival in ALS. Future longitudinal studies will be able to determine the viability of in vivo measures of p62 expression in identifying patients with poorer prognosis at presentation.

## Supplementary Material

nlad051_Supplementary_Data

## Data Availability

The datasets generated during this study are available from the corresponding author on reasonable request.
