# Supplementary material for: Survival in sporadic ALS is associated with lower p62 burden in the spinal cord
Source: J Neuropathol Exp Neurol. 2023 Jul 6;82(9):769–73. doi: 10.1093/jnen/nlad051 (PMC10440721; doi:10.1093/jnen/nlad051)
Supplement: nlad051_Supplementary_Data [file nlad051_supplementary_data.zip › Supplementary Table 1.docx]

**Supplemental Table 1.** Density (mean ± SEM) of cytoplasmic pTDP-43 inclusions in upper and lower motor neurons of ALS cases with a disease duration of <2 years (short survival) or 4-7 years (longer survival).

| **pTDP-43** | **Short survival** | **Longer survival** | **p value** |
| --- | --- | --- | --- |
| **Spinal cord** | | | |
| Total motor neurons/mm^2^ | 2.3 ± 1.2 | 1.1 ± 0.6 | **0.002** |
| Motor neurons with pTDP/mm^2^ | 1.6 ± 0.3 | 0.5 ± 0.1 | **0.001** |
| pTDP skein-like inclusions/mm^2^ | 0.6 ± 0.2 | 0.1 ± 0.05 | **0.02** |
| Round pTDP NCIs/mm^2^ | 0.2 ± 0.08 | 0.07 ± 0.03 | 0.1 |
| Diffuse pTDP NCIs/mm^2^ | 0.1 ± 0.07 | 0.04 ± 0.02 | 0.07 |
| Punctate pTDP NCIs/mm^2^ | 0.7 ± 0.2 | 0.3 ± 0.1 | 0.09 |
| **Hypoglossal nucleus** | | | |
| Total motor neurons/mm^2^ | 5.2 ± 3.1 | 4.0 ± 2.5 | 0.3 |
| Motor neurons with pTDP/mm^2^ | 2.5 ± 0.6 | 1.0 ± 0.3 | **0.02** |
| pTDP skein-like inclusions/mm^2^ | 1.3 ± 0.6 | 0.5 ± 0.3 | 0.2 |
| Round pTDP NCIs/mm^2^ | 0.2 ± 0.1 | 0.06 ± 0.04 | 0.2 |
| Diffuse pTDP NCIs/mm^2^ | 0.1 ± 0.06 | 0.1 ± 0.06 | 0.7 |
| Punctate pTDP NCIs/mm^2^ | 0.8 ± 0.3 | 0.3 ± 0.1 | 0.08 |
| **Motor cortex** | | | |
| Total motor neurons/mm^2^ | 0.04 ± 0.04 | 0.02 ± 0.02 | 0.09 |
| Motor neurons with diffuse pTDP/mm^2^ | 0.003 ± 0.002 | 0.001 ± 0.001 | 0.3 |

NCI: neuronal cytoplasmic inclusions.
