# Supplementary material for: Survival in sporadic ALS is associated with lower p62 burden in the spinal cord
Source: J Neuropathol Exp Neurol. 2023 Jul 6;82(9):769–73. doi: 10.1093/jnen/nlad051 (PMC10440721; doi:10.1093/jnen/nlad051)
Supplement: nlad051_Supplementary_Data [file nlad051_supplementary_data.zip › Rays redone Tan Supplementary Figure.pptx]

## Slide 1
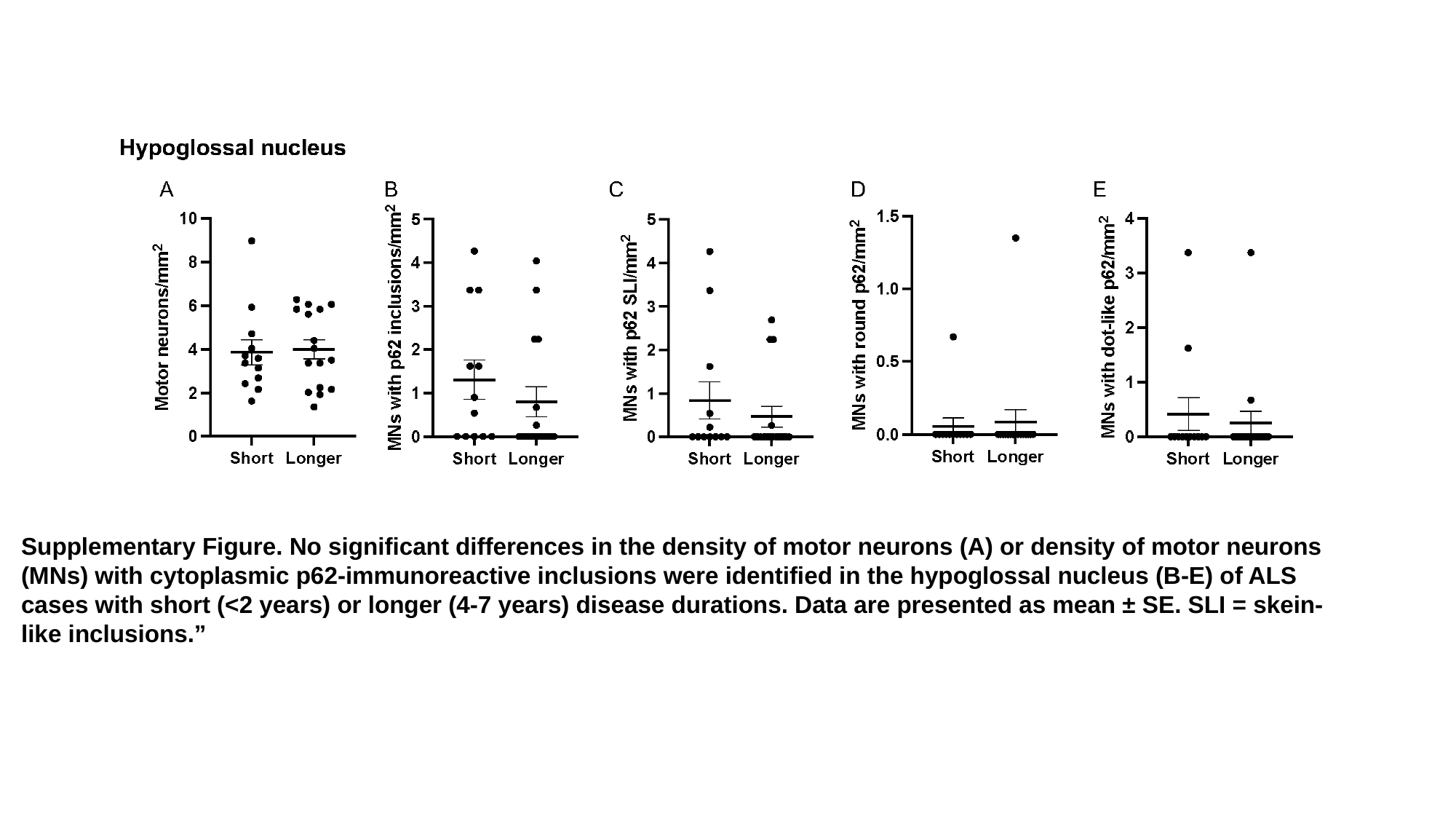

Supplementary Figure. No significant differences in the density of motor neurons (A) or density of motor neurons (MNs) with cytoplasmic p62-immunoreactive inclusions were identified in the hypoglossal nucleus (B-E) of ALS cases with short (<2 years) or longer (4-7 years) disease durations. Data are presented as mean ± SE. SLI = skein-like inclusions.”
